# Activation of protease-activated receptors (PARs)-1 and -2 promotes alpha-smooth muscle actin expression and release of cytokines from human lung fibroblasts

**DOI:** 10.14814/phy2.12295

**Published:** 2015-02-06

**Authors:** Nithiananthan Asokananthan, Rommel S Lan, Peter T Graham, Anthony J Bakker, Ana Tokanović, Geoffrey A Stewart

**Affiliations:** 1 School Pathology and Laboratory Medicine, University of Western Australia35 Stirling Highway, Crawley, Perth, WA, Australia; 2 School of Psychology and Clinical Sciences, Charles Darwin University, Ellengowan DriveCasuarina, Darwin, NT, Australia; 3 School of Anatomy, Physiology and Human Biology, University of Western Australia35 Stirling Highway, Crawley, Perth, WA, Australia

**Keywords:** Cell differentiation, cytokines, inflammation, lung

## Abstract

Previous studies have shown that protease-activated receptors (PARs) play an important role in various physiological processes. In the present investigation, we determined the expression of PARs on human lung fibroblasts (HLF-1) and whether they were involved in cellular differentiation and pro-inflammatory cytokine and prostaglandin (PGE_2_) secretion. PAR-1, PAR-2, PAR-3, and PAR-4 were detected in fibroblasts using RT-PCR, immunocytochemistry, and flow cytometry. Increased expression of PAR-4, but not other PARs, was observed in fibroblasts stimulated with phorbol myristate acetate. The archetypical activators of PARs, namely, thrombin and trypsin, as well as PAR-1 and PAR-2 agonist peptides, stimulated transient increases in intracellular Ca^2+^, and promoted increased α-smooth muscle actin expression. The proteolytic and peptidic PAR activators also stimulated the release of IL-6 and IL-8, as well as PGE_2_, with a rank order of potency of PAR-1 > PAR-2. The combined stimulation of PAR-1 and PAR-2 resulted in an additive release of both IL-6 and IL-8. In contrast, PAR-3 and PAR-4 agonist peptides, as well as all the PAR control peptides examined, were inactive. These results suggest an important role for PARs associated with fibroblasts in the modulation of inflammation and remodeling in the airway.

## Introduction

Bronchial asthma is a disease characterized by pulmonary inflammation and airway wall remodeling (Bousquet et al. 2000; Martinez and Vercelli 2013) and these features are major contributing factors toward the development of irreversible airway obstruction, bronchial hyperresponsiveness, and eventual decline of pulmonary function (Vignola et al. 2000; Chiappara et al. 2001; Fanta 2009). Although many cell types are involved in these processes, increasing evidence suggests that fibroblasts within the airway wall are primarily involved in airway wall remodeling and asthma. Increased numbers of subepithelial fibroblasts in the airways of asthmatic subjects have been reported (Holgate et al. 2000) and the number of fibroblasts has been positively correlated with the thickness of the basement reticular membrane (Holgate et al. 2000) as well as the severity of asthma (Benayoun et al. 2003). The increase in airway fibroblast proliferation may, in part, be due to increased concentrations of growth factors in the extracellular space. Transforming growth factor (TGF)-β, fibroblast growth factor, endothelin-1, and epidermal growth factor are predominantly derived from the airway epithelium (Chiappara et al. 2001) and have all been found to be elevated in asthmatic patients (Goldie and Henry 1999; Knight 2001). The role of these profibrotic mediators is to promote interstitial collagen deposition into the extracellular matrix, leading to thickening of the basement membrane (Holgate et al. 2000).

In addition, lung fibroblasts also proliferate, differentiate, and synthesize extracellular matrix components in response to Th2 cytokines such as IL-4 and IL-13 (James et al. 1989; Silvestri et al. 2001; Sabatini et al. 2002). These cytokines have also been shown to upregulate adhesion molecule expression on the surface of fibroblasts and promote inflammatory cell trafficking to the airway wall. Taken together, these findings suggest that lung fibroblasts may respond to various growth factors and profibrotic mediators in the extracellular space, stimulating cellular proliferation and augmenting the initial preinflammatory stimulus. Such structural changes are likely to increase airway wall thickening and stiffness, contributing to persistent airway obstruction and hyperresponsiveness observed in asthma (James et al. 1989; Saunders et al. 2009).

Interestingly, airway fibroblasts from asthmatic patients demonstrate an increased ability to differentiate into myofibroblasts (Michalik et al. 2009) which possess ultrastructural features intermediate between fibroblasts and smooth muscle cells. These cells are characterized by the presence of contractile proteins such as alpha-smooth muscle actin (α-SMA) (Darby et al. 1990; D'Andrea et al. 2001). Myofibroblasts are present in the asthmatic airways (Brewster et al. 1990; Holgate et al. 2000) and the number of these cells is increased upon bronchial allergen challenges in asthmatic subjects (Gizycki et al. 1997). In addition to matrix deposition, cytokine release, and expressing adhesion molecules, myofibroblasts are also likely to further contribute to luminal obstruction through contraction of the airway wall (Thannickal et al. 2004; Nihlberg et al. 2006).

In addition to profibrotic mediators and inflammatory cytokines, airway fibroblasts also respond to endogenous proteases such as thrombin and mast cell tryptase (Akers et al. 2000; Chambers et al. 2000). The effects of these proteases are, in part, mediated by a family of G-protein-coupled protease-activated receptors (PAR-1 to PAR-4). The extracellular portions of each PAR are cleaved by proteases, resulting in a new amino terminus which then binds to the second extracellular domain, initiating intracellular signaling and activation of cellular processes. Stimulation of PARs is associated with multiple signals transduction pathways, including inositol trisphosphate hydrolysis, increased cytosolic free Ca^2+^ concentration, and NF-κB expression (Macfarlane et al. 2001). In airway epithelial cells, activation of PARs stimulates the release of pro-inflammatory cytokines, suggesting that PARs promote tissue inflammation and repair (Asokananthan et al. 2002; Lan et al. 2002).

The precise role of PARs in fibroblasts function in the lung remains unclear, but there is evidence to show that activation of PAR-1 or PAR-2 promotes fibroblast proliferation (Akers et al. 2000; Shimizu et al. 2000; Frungieri et al. 2002) which may be dependent on the autocrine release of connective tissue growth factor (CTGF) (Chambers et al. 2000; Shimizu et al. 2000). In addition, PAR-1 or PAR-2 agonist peptides also induce cytoskeletal changes in these cells (Bogatkevich et al. 2001; Ge et al. 2003). These studies suggest that PAR activation in fibroblasts may promote airway wall remodeling (Akers et al. 2000; Chambers et al. 2000).

Whether activation of PARs results in inflammatory cytokine and prostaglandin E_2_ (PGE_2_) release from fibroblasts and promotes myofibroblast differentiation has not been examined in great detail. To this end, we examined the activation pathway of all four PARs and assessed the functional significance of PAR activation in human lung fibroblasts. In particular, we have focused on changes in intracellular Ca^2+^, cytokine and PGE_2_ secretions, and α-SMA expression.

## Materials and Methods

### Materials

Human lung fibroblast cells (HLF-1; American Tissue Culture Collection, Manassas, VA) were kindly provided by Professor Darryl Knight (School of Biomedical Sciences and Pharmacy, Newcastle University). Synthetic PAR agonist peptides, as well as control peptides, were synthesized with amidated C-termini (purity > 85 %) by Proteomics International Ltd. (Perth, WA, Australia). The sequences of the agonist and control peptides were, respectively: PAR-1, TFLLRN-NH_2_ and FTLLRN-NH_2_; PAR-2, SLIGKV-NH_2_ and LSIGKV-NH_2_; PAR-3, TFRGAP-NH_2_ and FTRGAP-NH_2_; and PAR-4, GYPGQV-NH_2_ and GYPGVQ-NH_2_. As it has previously been shown that the human PAR-1 agonist peptide, SFLLRN, could cross-activate PAR-2, the more specific xenopus-human hybrid PAR-1 agonist peptide TFLLRN-NH_2_ was used in this study (Hollenberg et al. 1997). Thrombin was purchased from CSL Limited (Melbourne, VIC, Australia) and Sigma Chemical Co. (St Louis, MO). Tissue culture and molecular biology reagents were purchased from Invitrogen Corp. (Carsbad, CA). Unless otherwise stated, other general chemicals were purchased from either BDH (Kilsyth, Vic., Australia) or from Sigma Chemical Co.

### Protease activity

Trypsin activity was confirmed using the active site titrant, p-nitrophenyl p'-guanidino benzoate**,** as described previously (Chase and Shaw 1969). Thrombin activity was determined using the thrombin substrate N- benzoyl-D_L_-arginine p-nitroanilide (King et al. 1996). Thrombin, obtained from CSL Limited (0.5 nmol L^−1^ p-nitroaniline released min^−1^ mg^−1^), was approximately 50-fold less active than that purchased from Sigma Chemical Co. (23.4 p-nitroaniline released min^−1^ mg^−1^). Thrombin from the former source was used in cell culture experiments, whereas the latter was used in Ca^2+^ flux studies.

### Human lung fibroblast cell culture and PAR stimulation

HLF-1 fibroblasts were cultured in 75 cm^2^ tissue culture flasks (Nalge Nunc International, Naperville, IL), and grown in DMEM medium supplemented with 10 % (v/v) fetal calf serum (FCS), and antibiotics. Cells from the flasks were trypsinized and seeded into 24-well tissue culture plates at a density of 2.5 × 10^5^ per well for 24 h, and serum-starved overnight. Serum-deprived HLF-1 cells were exposed to varying concentrations of thrombin (0.005–50 U mL^−1^) or trypsin (1.25 × 10^−4^–1.25 nmol L^−1^), as higher concentrations of these peptidases caused detachment and cellular death (data not shown). HLF-1 cells were also stimulated with PAR peptides (100–500 μmol L^−1^) or phorbol myristate acetate (PMA, 10 ng mL^−1^). In some experiments, cells were incubated with indomethacin (10 μmol L^−1^) prior to stimulation to assess the role of cyclooxygenase in PGE_2_ release. Aliquots (100 μL) of cell culture supernatants were removed at various time points (0, 3, 6, 12, 18, and 24 h) after stimulation, centrifuged at 12,000 ×* g* for 5 min at 4°C, and stored at −20°C until ready for cytokine quantitation. Cells stimulated in this way showed greater than 85 % viability at the conclusion of the experiments, as determined by trypan blue exclusion and lactate dehydrogenase assays (not shown).

### Reverse transcription–polymerase chain reaction (RT-PCR)

Total RNA was prepared from HLF-1 fibroblasts using Tri-Reagent according to the manufacturer's instructions (Molecular Research Center, Cincinnati, OH). Briefly, cells were lysed and the homogenates were transferred to 1.5 mL microcentrifuge tubes. Chloroform (200 μL) was added to each tube, shaken by hand, and left at room temperature (RT) for 10 min. Tubes were then centrifuged at 13,000 *× g* for 15 min at RT, after which the aqueous phases were transferred to new tubes containing 0.5 mL of isopropanol. The tubes were left overnight at −20°C, and then centrifuged at 13,000 *× g* for 15 min. The supernatants were discarded, and 1 mL 75% (v/v) ethanol was added to the pelleted RNA in each tube. The pellets were resuspended and tubes centrifuged at 13,000 *× g* for 8 min, after which the supernatants were discarded and the pellets air-dried, and dissolved in 10 μL of diethyl pyrocarbonate (DEPC)-containing water. For reverse transcription, RNA was primed with 250 ng of oligo-dT_12-18_ primer, and reversed transcribed in a mastermix totaling 50 μL, containing 7.5 mmol L^−1^ MgCl_2_, 0.4 mmol L^−1^ of each dNTP, 10U RNase inhibitor, and 2.5 U avian myeloblastosis virus reverse transcriptase (Promega, Madison, WI). The mixture was incubated at 42°C for 60 min, and terminated by heating at 90°C for 2 min. The synthesized first strand cDNA was stored at −20°C.

Forward and reverse primers used for amplifying human PARs were prepared commercially based on published sequence data (Wan et al. 2001); PAR-1 sense 5′-TGTGAACTGATCATGTTTATG-3′, antisense 5′-TTCGTAAGATAAGAGATATGT-3′, (PCR product, 708 bp); PAR-2 sense 5′- AGAAGCCTTATTGGTAAGGTT-3′, antisense 5′-AACATCATGACAGGTCGTGAT-3′ (PCR product, 582 bp); PAR-3 sense 5′-CTGATACCTGCCATCTACCTCC-3, antisense 5′- AGAAAACTGTTGCCCACACC-3′, (PCR product, 382 bp); PAR-4 sense 5′- ATTACTCGGACCCGAGCC-3′, antisense 5′-TGTAAGGCCCACCCTTCTC-3′ (PCR product, 392 bp). Amplification of GAPDH, with the sense and antisense primer pair 5′- CCCATCACCATCTTCCAGGAGC-3′ and 5′-CCAGTGAGCTTCCCGTTCAGC-3′ (PCR product, 471 bp), acted as an internal control (Prime et al. 2000). For the polymerase chain reaction, 1.5 μL cDNA was mixed in a reaction vial containing 2.5 pmol L^−1^ of each forward and reverse primer, 0.2 mmol L^−1^ of each dNTP, 2 mmol L^−1^ MgCl_2_, 0.5 U Platinum Taq DNA polymerase, 1.2 μL of 10 × Taq DNA polymerase buffer, and made up to 12 μL using DEPC water. The conditions for amplification were as follows: for PAR-1 and PAR-2, 94°C for 45 sec, 55°C for 45 sec, 72°C for 2 min and 30 sec, for 35 cycles; for PAR-3, PAR-4 and GAPDH, 94°C for 45 sec, 65°C for 45 sec, 72°C for 2 min and 30 sec, for 35 cycles. Electrophoresis was performed using 2% (w/v) analytical grade agarose gels which were subsequently stained with ethidium bromide, and visualized under a UV transilluminator. Densitometric analysis was performed using NIH Image software (National Institute of Health, Bethesda, MD).

### Immunocytochemistry and fluorescence/confocal microscopy

Immunolocalization of PARs on fibroblasts was performed using cells cultured on glass slides. The cells were rinsed with PBS and fixed in 4 % (v/v) paraformaldehyde in PBS for 30 min at RT. The cells were permeabilized with 0.2 % Triton X (v/v) in PBS for 5 min at RT, and rinsed in PBS. After blocking the cells in 20 % normal horse serum, the fibroblasts were incubated with antibodies raised against individual PARs, including mouse monoclonal antibodies (Santa Cruz Biotechnology Inc., Santa Cruz, CA) raised against PAR-1 (ATAP2 sc-13503) and PAR-2 (SAM11; sc-13504), as well as rabbit antibodies (commercially prepared by Chiron, Clayton, Australia) raised against PAR-3 (_37_TLPIKTFRGAPPNSFEEFP_55_) and PAR-4 (_28_EDDSTPSLLPAPRGYPGQV_39_). Equivalent dilutions of isotype-matched mouse and rabbit antibodies (Dako, Via Real Carpinteria, CV) were also used as appropriate controls. The primary antibodies were incubated overnight at 4°C. Following two washes in PBS, the cells were incubated with appropriate Alexa 488-conjugated secondary antibodies (Molecular Probes, Eugene, OR) for 60 min at RT, rinsed twice in PBS, mounted in a nonfluorescent mounting medium, and kept in the dark until ready for viewing using fluorescence (TE2000-U; Nikon Corp., Chiyoda-ku, Tokyo, Japan) or confocal microscopy (Biomedical and Imaging Analysis Facility, UWA, Perth, WA, Australia).

### Flow cytometry

Fibroblasts cultured in 24-well tissue culture plates were removed by gentle pipetting the cells previously incubated in 8 mmol L^−1^ EDTA in PBS at 4°C for 5 min. The cells were centrifuged (500 *× g,* 6 min), fixed with 4 % (v/v) paraformaldehyde in PBS for 30 min at RT, and blocked in 10 % (v/v) normal horse serum (30 min, RT). Specific anti-PAR antibodies were diluted in PBS or PBS with 0.1 % (w/v) saponin, to assess cell surface and intracellular PAR staining, respectively. The primary antibodies, as well as the isotype-matched control antibodies, were incubated overnight at 4°C. Following two washes in PBS, the cells were incubated with appropriate biotin-conjugated secondary antibodies for 2 h at RT, and washed twice in PBS. The cells were then incubated with phycoerythrin (PE)-conjugated streptavidin (BD Bioscience Pharmingen, San Diego, CA) for 2 h at RT in the dark, washed twice in PBS, and stored in the dark at 4°C. Cells were assayed on a FACS Caliber (Biomedical and Imaging Analysis Facility, UWA), and files of 10^4^ events were analyzed using FlowJo software (Tree Star Inc., Ashland, OR).

### Detection of IL-6 and IL-8 by ELISA

Supernatants from stimulated fibroblasts were assayed for IL-6 and IL-8 using ELISA. Briefly, 96-well plates were coated with 100 μL of the appropriate antibody (500 ng mL^−1^ in 0.1 M NaHCO_3_/NaCO_3_ buffer) and incubated overnight at 4°C. The plates were then washed three times with washing buffer (PBS containing 0.5 % (v/v) Tween-20) and blocked with 100 μL of washing buffer containing 1 % (w/v) BSA at RT for 1 h. The plates were washed three times with washing buffer before 100 μL aliquots of each sample, or cytokine standards, were added to the wells. The plates were incubated overnight at 4°C and then washed three times. The appropriate biotinylated secondary antibodies were then added to each well at RT for 1 h and washed three times. Streptavidin-horseradish peroxidase conjugates were added to each well, incubated at RT for 30 min, and the plates then washed three times. Finally, 100 μL of peroxidase substrate (K-Blue ELISA substrate, Graphic Scientific, Brisbane, QLD, Australia) was added to each well, and reactions terminated by addition of 1 M phosphoric acid. Optical densities were determined using a microplate reader (Spectramax 250; Molecular Devices Co., Sunnyvale, CA) at 450 nm, and cytokine concentrations determined by interpolation from the standard curve using the SoftMax-Pro software (Molecular Devices Co.), and expressed as pg per 2.5 × 10^5^ viable cells.

### Detection of PGE_2_ by ELISA

PGE_2_ was measured using a competitive PGE_2_ enzyme immunoassay (Cayman Chemical, Ann Arbor, MI) as described previously (Ge et al. 2003). PGE_2_ released was expressed as pg per 2.5 × 10^5^ viable cells.

### Measurement of cytosolic Ca^2+^

Changes in cytosolic Ca^2+^ concentration were measured using the membrane permeable fluorescent Ca^2+^ indicator, Indo-1 (Teflabs, Austin, TX), as described previously (Asokananthan et al. 2002). In brief, cells were grown on coverslips to 80 % confluency and loaded with RPMI containing 6 μmol L^−1^ of Indo-1 and 0.1 % (w/v) Pluronic F-127 (Molecular Probes Inc., Eugene, OR) for 45 min at RT. After incubation, cells were washed with two changes of RPMI to remove the excess dye prior to challenges with PAR activators. The ratio of fluorescence emission at 405 and 490 nm was measured using a spectrophotometer (Cairn Research Ltd, Faversham, Kent, U.K.) attached to an inverted microscope (Nikon Corp.), configured for epifluorescence. The excitation wavelength of 340 nm was provided by a variable monochromator system (Cairn Research Ltd). The ratio of the emission intensities was used as a measure of changes in cytosolic Ca^2+^.

### Statistical analyses

Unless stated otherwise, data were expressed as mean ± standard error of the mean (SEM). Statistically significant differences between means were determined using the unpaired Student's *t*-test. *P* values less than 0.05 were considered statistically significant.

## Results

### Expression of PARs in HLF-1 human lung fibroblasts

**Figure 1:**
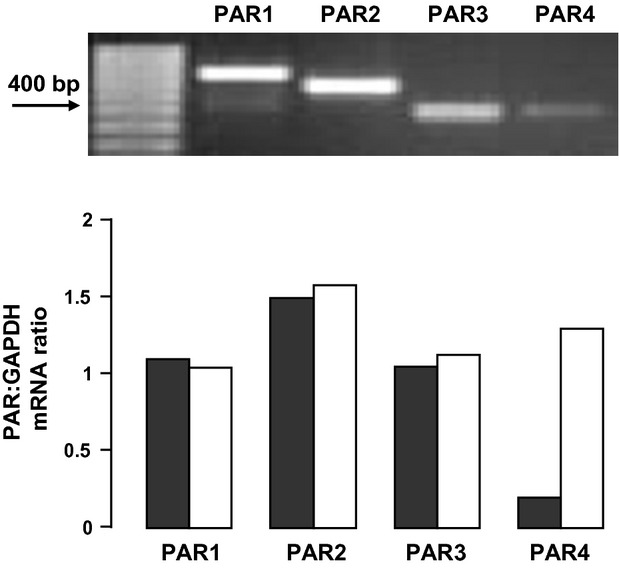
RT-PCR analysis of PAR mRNA expression in HLF-1 cells. RNA was extracted, reverse transcribed, and amplified using gene-specific primers. Amplicons were then separated on 2% (w/v) agarose gels, stained with ethidium bromide, and viewed under a UV transilluminator. Lanes depict (from left to right, top panel) 100 bp DNA ladder, and PAR-1 (708 bp), PAR-2 (582 bp), PAR-3 (382 bp), and PAR-4 (392 bp), from unstimulated HLF-1 cells. PAR mRNA expression was also determined from fibroblasts stimulated with either vehicle, or PMA for 24 h (bottom panel). Data presented represent the mean ratio of PAR:GAPDH mRNA expression derived from two different experiments.

**Figure 2:**
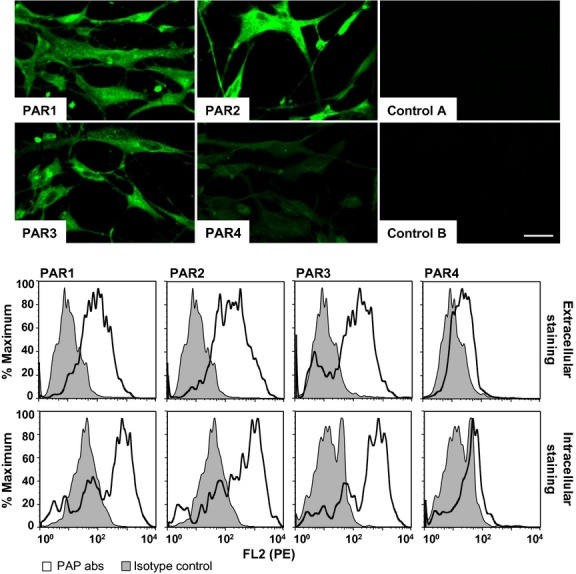
Immunocytochemistry (top panels) and FACS analyses (bottom panels) of PAR protein expression in HLF-1 cells. The fibroblasts were cultured on glass slides and stained with anti-PAR-1, PAR-2, PAR-3, and PAR-4 antibodies. Cells were also stained with appropriate mouse (control A) and rabbit (control B) isotype control antibodies. Bar = 20 μm (top panel). For FACS analyses, cells were similarly stained with PAR antibodies, or appropriate isotype controls (bottom panel). Cells were stained in the absence (extracellular staining) or presence of 0.1% (w/v) saponin (intra- and extracellular staining). Data presented are representative of experiments performed in duplicates on three separate occasions.

mRNA for PAR-1, PAR-2, PAR-3, and PAR-4 was detected by RT-PCR in HLF-1 fibroblasts, and the sizes of each amplicon detected corresponded to those previously reported (Ge et al. 2003; Jin et al. 2003) (Fig.1, upper panel). The intensity of the PAR-4 amplicon was less than that observed for PAR-1, PAR-2, or PAR-3, but treatment of cells with PMA (10 ng ml^−1^ for 24 h) upregulated PAR-4 expression, but not other PARs (Fig.1, lower panel). In addition, HLF-1 cells were shown to express PAR proteins as judged by both immunocytochemistry and flow cytometry (Fig.2). The intensity of immunoreactive PAR-1, PAR-2, and PAR-3 was greater than that observed using with PAR-4. Flow cytometry showed expression of PARs on the surface as well as in the cytosol.

### Changes in intracellular Ca^2+^ induced by peptidases and PAR peptides in HLF-1 cells

**Figure 3:**
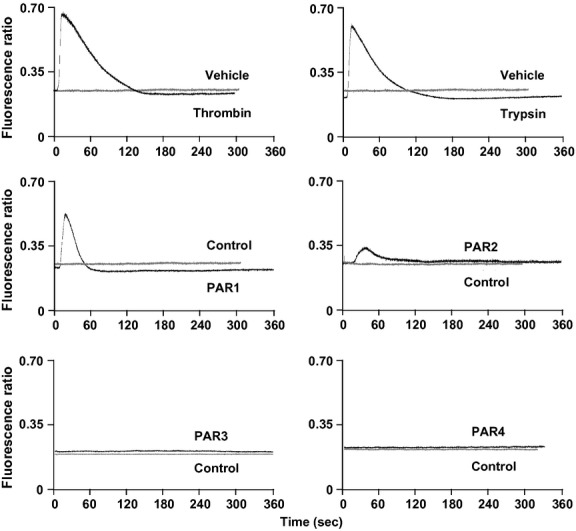
The effects of PAR activation on intracellular Ca^2+^ in HLF-1 cells. The fibroblasts were loaded with Indo-1, and stimulated with thrombin (10 U mL^−1^), trypsin (0.125 nmol L^−1^), or vehicle (saline). Indo-1-loaded HLF-1 cells were also stimulated with PAR agonist peptides (100 μmol L^−1^) or appropriate scrambled control peptides (Control). Responses were measured over 360 s, and expressed as a fluorescence ratio (405/490 nm). Data presented are representative of experiments performed on two to three separate occasions.

**Figure 4:**
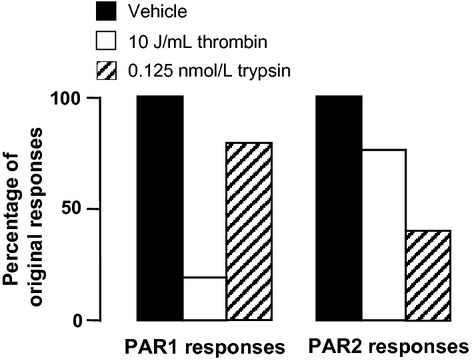
Effects of PAR agonist peptides in protease-desensitized HLF-1 cells. HLF-1 cells were loaded with Indo-1, and exposed to vehicle, 10 U mL^−1^ thrombin or 0.125 nmol L^−1^ trypsin. Upon reestablishing a stable baseline after the initial stimulus (approximately 300 s), cells were then activated with either PAR-1 or PAR-2 agonist peptides (100 μmol L^−1^). The increase in intracellular Ca^2+^ induced by the PAR agonist peptides in protease-pretreated cells were expressed as a percentage of the effects in cells pretreated with vehicle (saline). Data presented are the mean responses from experiments conducted on two separate occasions.

Both thrombin and trypsin induced changes in intracellular Ca^2+^ in HLF-1 cells, as judged by increases in fluorescence ratio (Fig.3). In addition, treatment of cells with either PAR-1 or PAR-2 agonist peptides also induced changes in intracellular Ca^2+^ (Fig.3). Intracellular Ca^2+^ increased within a few seconds of exposure to proteases and PAR agonist peptides, and gradually returned to baseline within 120 sec. PAR-3 and PAR-4 agonist peptides, as well as the controls peptides for each of the four PARs, were inactive. In addition, PAR-1 and PAR-2 agonist peptide-induced changes in Ca^2+^ were significantly attenuated when cells were previously challenged with thrombin, and trypsin, respectively. The effects of the PAR-1 and PAR-2 agonist peptide were slightly reduced in cells prestimulated with trypsin and thrombin, respectively (Fig.4).

### Induction of α-SMA expression in HLF-1 cells by proteases and PAR peptides

**Figure 5:**
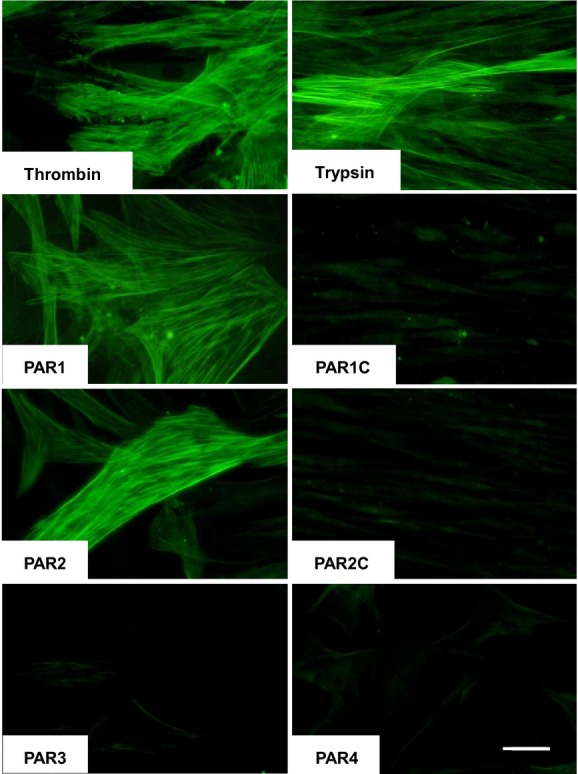
Detection of α-smooth muscle actin expression in HLF-1 cells using immunocytochemistry. The fibroblasts were stimulated with 3 U mL^−1^ thrombin, 12.5 pmol L^−1^ trypsin, 400 μmol L^−1^ PAR agonist peptides (PAR-1, PAR-2, PAR-3, and PAR-4), or appropriate control peptides for PAR-1 (PAR1C) and PAR-2 (PAR2C). The cells were subsequently fixed, stained using an anti-α-smooth muscle actin antibody, and visualized using fluorescence microscopy. Bar = 50 μm.

Stimulating HLF-1 cells with thrombin, trypsin, and PAR-1 and PAR-2 agonist peptides for 24 h clearly induced the expression of α-SMA in HLF-1 cells. In contrast, cells stimulated with PAR-3 and PAR-4 agonist peptides, or PAR control peptides, expressed no detectable α-SMA (Fig.5).

### Thrombin and trypsin induce cytokine release from HLF-1 cells

**Figure 6:**
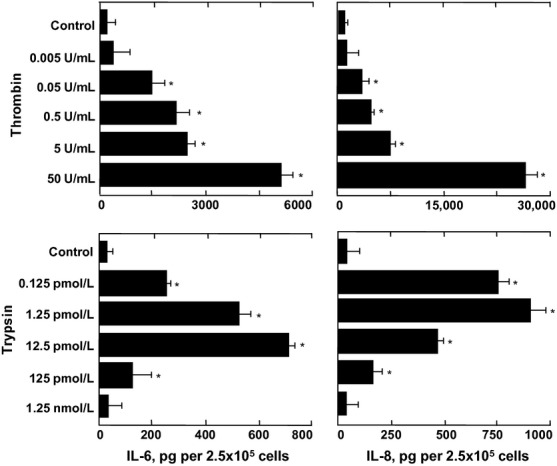
The effects of trypsin and thrombin on cytokine release from HLF-1 cells. Fibroblasts were cultured overnight in serum-free medium then stimulated with increasing concentrations of either thrombin (upper panels) or trypsin (lower panels). IL-6 and IL-8 in cell culture supernatants were quantitated using ELISA, and data expressed as mean pg ± SEM per 2.5 × 10^5^ cells from three independent experiments performed in quadruplicate. *P < 0.05 comparing the differences in mean cytokine release from control supernatants with those from thrombin- and trypsin-treated cells.

Thrombin stimulated the release of IL-6 and IL-8 from HLF-1 cells after 24 h in a concentration-dependent manner (Fig.6). The maximal release of cytokines occurred when the cells were stimulated with 50 U mL^−1^ thrombin. Similarly, trypsin concentration dependently stimulated the release of IL-6 and IL-8 from HLF-1 cells after 24 h. Maximal release of IL-6 and IL-8 release was observed when the concentration of trypsin used was at 12.5 pmol L^−1^ and 1.25 pmol L^−1^, respectively. The maximal release of IL-6 and IL-8 induced by thrombin was, respectively, 7-fold and 29-fold greater than that induced by trypsin (Fig.6).

### PAR agonist peptides induce cytokine release from HLF-1 cells

**Figure 7:**
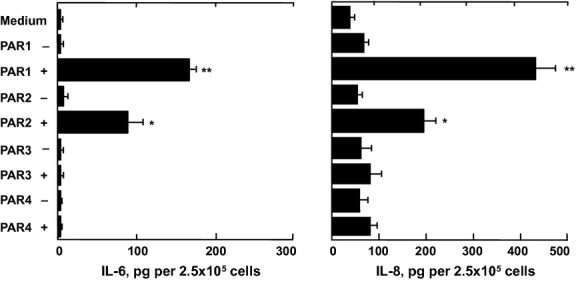
The effects of PAR peptides on cytokine release from HLF-1 cells. Fibroblasts were cultured overnight with serum-free medium then stimulated with 400 μmol L^−1^ of PAR agonist peptides (+) or control scrambled peptides (−) for 24 h. Cytokine release were determined by ELISA, and data expressed as mean pg ± SEM per 2.5 × 10^5^ cells from three independent experiments performed in quadruplicate. *P < 0.05, **P < 0.01 comparing the differences in mean cytokine release from control supernatant and those from PAR peptide-treated cells.

**Figure 8:**
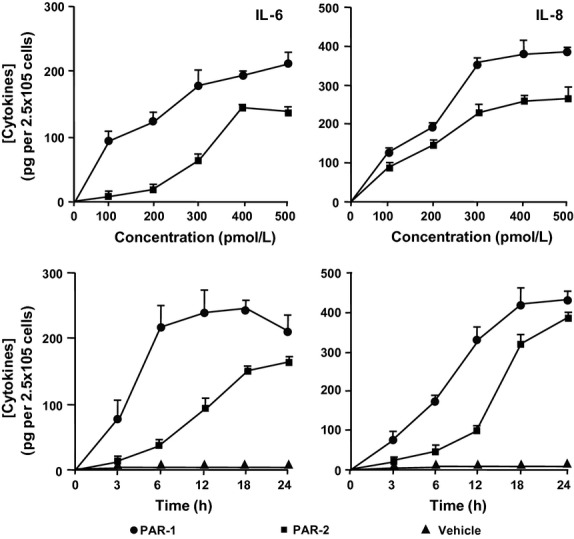
The effects of PAR-1 and PAR-2 agonist peptides on cytokine release from HLF-1 cells. Fibroblasts were cultured overnight with serum-free medium, then stimulated with either different concentrations of the PAR-1 or PAR-2 agonist peptides for 24 h (top panels). Fibroblasts were also stimulated with 400 μmol L^−1^ PAR peptides, or vehicle, for different durations (bottom panels). IL-6 (left panels) and IL-8 (right panels) release was determined by ELISA, and expressed as mean ± SEM per 2.5 × 10^5^ cells from three independent experiments performed in quadruplicate.

Exposure of HLF-1 cells to 400 μmol L^−1^ PAR-1 or PAR-2 agonist peptides for 24 h also stimulated the secretion of IL-6 and IL-8 (Fig.7). The release of IL-6 and IL-8 induced by the PAR-1 agonist peptide was approximately 2-fold greater than that induced by the PAR-2 agonist peptide. The PAR-3 and PAR-4 agonist peptides, as well as the control peptides for each of the four PARs, did not significantly modulate basal cytokine secretion. The PAR-1 and PAR-2 agonist peptides concentration dependently induced the release of cytokines, with maximal cytokine release obtained when 300–500 μmol L^−1^ of agonist peptides were used (Fig.8). The release of cytokines induced by 400 μmol L^−1^ of PAR agonist peptides was also time dependent (0–24 h), with maximal IL-6 and IL-8 release observed 6 and 18 h after peptide stimulation, respectively (Fig.8).

### HLF-1 cells respond to additive combinations of PAR agonist peptides

**Figure 9:**
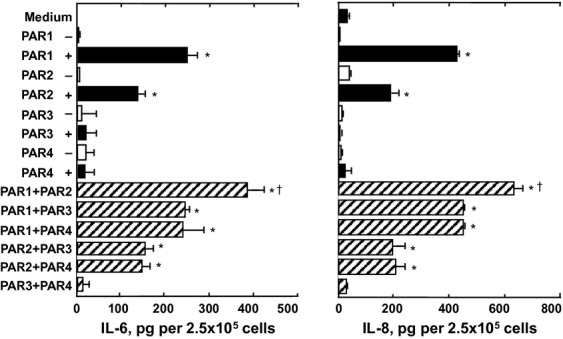
The additive effect of PAR agonist peptides on cytokine secretion from HLF-1 cells. Fibroblasts were cultured in serum-free medium overnight, and then treated with PAR agonist peptide alone (400 μmol L^−1^ for 24 h) or in combination with other PAR peptides. IL-6 and IL-8 release was determined by ELISA, and data expressed as mean pg ± SEM per 2.5 × 10^5^ cells from three independent experiments performed in quadruplicate. *P < 0.05 comparing the differences in mean cytokine release from control supernatant. ^†^*P *< 0.05 comparing the differences in mean cytokine release from the effects of PAR-1 or PAR-2 agonist peptide alone.

Exposure of HLF-1 cells to PAR-2 agonist peptide in combination with PAR-1 agonist peptides (each at 400 μmol L^−1^) resulted in the statistically significant additive release of both IL-6 and IL-8 (Fig.9). Combinations of PAR-3 or PAR-4 agonist peptides with PAR-1 or PAR-2 agonist peptides did not cause any additive or synergistic cytokine release (Fig.9).

### PAR agonist peptides induce PGE_2_ release from HLF-1 cells

**Figure 10:**
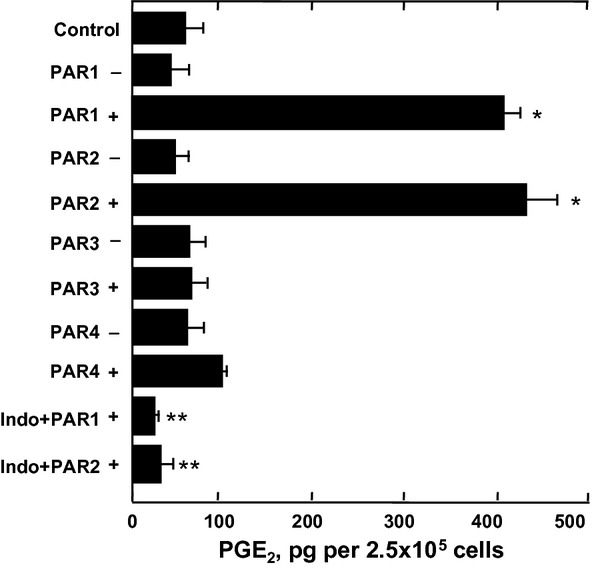
The effect of PAR peptides on PGE_2_ release from HLF-1 cells. Fibroblasts were cultured in serum-free medium overnight prior to stimulation with 400 μmol L^−1^ PAR agonist (+), or control (−), peptides for 24 h. In some experiments, cells were pretreated with medium containing indomethacin (Indo, 10 μmol L^−1^) before stimulation with PAR-1 or PAR-2 agonist peptides. PGE_2_ in supernatants was determined by enzyme immunosorbent assay, and the data expressed as mean pg ± SEM per 2.5 × 10^5^ cells from three independent experiments performed in quadruplicate. *P < 0.05 comparing the differences in mean cytokine release to medium control. **P < 0.05 comparing the differences in mean cytokine release to the effects of the agonist peptide in the absence of indomethacin.

Stimulation of HLF-1 fibroblasts with PAR-1 and PAR-2 agonist peptides significantly increased the release of PGE_2_ by these cells. However, neither PAR-3 or PAR-4 agonist peptides nor the control peptides had any effect (Fig.10). PAR agonist peptide-induced PGE_2_ release was significantly inhibited by pretreating the cells with 10 μmol L^−1^ indomethacin (Fig.10).

## Discussion

We have been able to demonstrate the expression of PAR-1, PAR-2, PAR-3, and PAR-4 using RT-PCR, immunocytochemistry, and flow cytometry. Previous studies by Sokolova et al. (2005) have demonstrated detection of PAR expression using immunohistochemistry, while others have examined the expression of PAR mRNA levels using RT-PCR (Ramachandran et al. 2006; Ortiz-Stern et al. 2012). In line with previous observations (Ramachandran et al. 2007) PAR-4 did not appear to be abundantly expressed on human lung fibroblasts unlike the other PAR receptors. We also showed that PMA, an activator of protein kinase C (Saitoh and Dobkins 1986) increased PAR-4 mRNA transcripts in human lung fibroblasts. These findings, coupled with the lack of functional responses to PAR-4 agonist peptides in naive HLF-1 cells, suggest that PAR-4 may not play a significant role in protease modulation of lung fibroblast function during tissue homeostasis. However, inflammatory trauma and pro-inflammatory cytokines such as TNFα and IL-1β may increase PAR expression in various cells including skeletal muscle, airway epithelium, and endothelial cells (Nystedt et al. 1996; Mbebi et al. 2001; Lan et al. 2004).

The activation of PARs by thrombin and trypsin, as well as the peptide activators of PAR-1 and PAR-2, was accompanied by changes in intracellular Ca^2+^ in lung fibroblasts which is consistent with PAR-mediated signaling via Gq. As some PAR-1 peptides have previously been shown to cross-activate other PARs (Howell et al. 2001), cross-desensitization experiments between PAR-1 and PAR-2 were conducted, and it was demonstrated that responses to the PAR-1 (TFLLRN) and PAR-2 (SLIGRL) agonist peptides were significantly attenuated when the cells were pretreated with thrombin and trypsin, respectively.

α-SMA expression is an indicator of actively proliferating fibroblasts, and a distinctive marker of myofibroblasts differentiation. We now demonstrate that the agonist peptides to both PAR-1 or PAR-2, but not PAR-3 nor PAR-4, stimulate α−SMA expression in human lung fibroblasts. Similar results have been obtained by Bogatkevich (Bogatkevich et al. 2009) demonstrating that inhibition of thrombin leads to prevention of α-SMA expression in human lung fibroblasts in a PAR-1-dependent manner while Borensztajn's work has indicated that PAR-2 activation can also lead to α-SMA expression in vivo (Borensztajn et al. 2010). Increased numbers of α-SMA- expressing myofibroblasts have previously been reported in the airways of subjects with asthma and idiopathic pulmonary fibrosis (Brewster et al. 1990; Saunders et al. 2009). Although important in wound healing (Thannickal et al. 2004), myofibroblasts are the predominant pulmonary cells contributing toward increasing submucosal thickness and collagen deposition in the airway wall of asthmatic subjects (Roche et al. 1989; Brewster et al. 1990; Ward and Hunninghake 1998). In asthma, both the expression of PARs (Knight et al. 2001), as well as the concentrations of PAR activators such as thrombin (Gabazza et al. 1999), are increased in the airways, and thrombin inhibitors have been shown to attenuate airway wall remodeling in a murine model of bleomycin-induced pulmonary fibrosis (Howell et al. 2001). Our findings suggest that PAR-differentiated myofibroblasts are likely to promote tissue repair processes, but in chronic airway inflammatory disorders, myofibroblasts may adversely contribute to airway hyperresponsiveness and irreversible airway wall remodeling through increased collagen secretion and cellular proliferation (Darby et al. 1990; Phan 2002).

There is increasing evidence to suggest that fibroblasts play an important role in lung inflammatory processes. In this study, we showed that the activation of both PAR-1 and PAR-2 stimulated the release of the immunomodulatory mediators IL-6, IL-8, and PGE_2_, with the PAR-1 agonist peptide exhibiting greater potency than PAR-2. Mediator release induced by either PAR- 1 and PAR-2 agonist peptides was shown to be additive, with the secreted levels of IL-6 and IL-8 being significantly higher when the cells were stimulated with both PAR-1 and PAR-2 agonist peptides in combination, compared to the singular effects of PAR-1 or PAR-2 agonist peptides.

These findings were similar to our previous observations in airway epithelial cells (Asokananthan et al. 2002), and along with the present data using cross-desensitization Ca^2+^ assays, we confirmed that the PAR agonist peptides used in this study were acting through distinct receptors, PAR-1 and PAR-2. Similar results have been obtained in a study using thoracotomy samples, where PAR-2 agonists upregulated granulocyte colony-stimulating factor, IL-8 and VCAM-1 in bronchial fibroblasts (Ramachandran et al. 2006), while another group found thrombin stimulation of human lung fibroblasts via PAR-1 promoted secretion of CCL2, a chemotactant which acts on monocytes, natural killer cells, T cells, and fibrocytes (Ortiz-Stern et al. 2012). Sokolova and colleagues also found similar effects of trypsin and thrombin stimulation of lung fibroblasts on calcium flux and PGE2 (Sokolova et al. 2005).

Interestingly, both the PAR-3 and PAR-4 agonist peptides were inactive. Indeed, it was previously demonstrated that PAR-3 is not functionally coupled to intracellular signal transduction pathway, but rather functions as a cofactor for the thrombin-sensitive receptor, PAR-4 (Nakanishi-Matsui et al. 2000). However, as PAR-4 is not abundantly expressed in human lung fibroblasts, the functional significance of PAR-3 in these cells is currently not known. It is of interest to note that the PAR-3 peptide may have other biological effects, such as stimulating intracellular Ca^2+^ changes in rat astrocytes and human vascular smooth muscle (Wang et al. 2002; Bretschneider et al. 2003) and there is recent evidence to suggest that it may cross-activate PAR-1 and PAR-2 (Hansen et al. 2004).

Proteases such as thrombin and trypsin detected in the airway may be expressed by resident pulmonary cells. For example, the expression of immunoreactive thrombin in alveolar macrophages has been reported (Howell et al. 2002), while trypsin has been detected in the respiratory epithelium (Cocks et al. 1999; Danahay et al. 2001; Cederqvist et al. 2003; Jin et al. 2003; Miki et al. 2003). Furthermore, it is established that the concentrations of these proteases are elevated in the bronchoalveolar lavage fluid in diseases such as asthma (Gabazza et al. 1999; Terada et al. 2004). Based on these findings, PAR activation by secreted proteases on lung fibroblasts is likely to have profound implications on the pathogenesis of asthma. In addition to cellular proliferation (Trejo et al. 1996; Akers et al. 2000) and pro-collagen secretion (Chambers et al. 1998), the simultaneous activation of PAR-1 and PAR-2 on lung fibroblasts is likely to have an additive effect on the inflammatory process in asthma. Furthermore, the differentiation of these cells into myofibroblasts via PAR activation is also likely to compound the disease process toward irreversible airway wall remodeling.

In conclusion, we report the presence of PAR-1, PAR-2, PAR-3, and PAR-4 in human airway fibroblasts. PAR-1 and PAR-2 expressed by these cells are functionally responsive to thrombin and trypsin, respectively. As PAR subtypes exhibit varying activation sensitivities to different proteases, the expression of multiple PARs within a single population of cells may discriminate different proteases in the extracellular space (Shapiro et al. 2000; Wang et al. 2002). Moreover, the activation of PAR-1 or PAR-2 in airway fibroblasts promotes myofibroblast differentiation (as indicated by increased expression of α-SMA expression) and the release of immunomodulatory mediators such as IL-6, IL-8, and PGE_2_. The release of cytokines induced by PAR agonist peptides was both time and concentration dependent. Our findings are strongly suggestive that PAR activation is likely to perpetuate airway inflammation and tissue remodeling, two features which are present in airway disorders such as asthma.

## Conflict of Interest

None of the authors have a financial relationship with a commercial entity that has an interest in the subject of this manuscript.
